# High Throughput Microplate Respiratory Measurements Using Minimal Quantities Of Isolated Mitochondria

**DOI:** 10.1371/journal.pone.0021746

**Published:** 2011-07-25

**Authors:** George W. Rogers, Martin D. Brand, Susanna Petrosyan, Deepthi Ashok, Alvaro A. Elorza, David A. Ferrick, Anne N. Murphy

**Affiliations:** 1 Seahorse Bioscience, North Billerica, Massachusetts, United States of America; 2 Buck Institute for Age Research, Novato, California, United States of America; 3 Department of Pharmacology, University of California San Diego, La Jolla, California, United States of America; Instituto de Química - Universidade de São Paulo, Brazil

## Abstract

Recently developed technologies have enabled multi-well measurement of O_2_ consumption, facilitating the rate of mitochondrial research, particularly regarding the mechanism of action of drugs and proteins that modulate metabolism. Among these technologies, the Seahorse XF24 Analyzer was designed for use with intact cells attached in a monolayer to a multi-well tissue culture plate. In order to have a high throughput assay system in which both energy demand and substrate availability can be tightly controlled, we have developed a protocol to expand the application of the XF24 Analyzer to include isolated mitochondria. Acquisition of optimal rates requires assay conditions that are unexpectedly distinct from those of conventional polarography. The optimized conditions, derived from experiments with isolated mouse liver mitochondria, allow multi-well assessment of rates of respiration and proton production by mitochondria attached to the bottom of the XF assay plate, and require extremely small quantities of material (1–10 µg of mitochondrial protein per well). Sequential measurement of basal, State 3, State 4, and uncoupler-stimulated respiration can be made in each well through additions of reagents from the injection ports. We describe optimization and validation of this technique using isolated mouse liver and rat heart mitochondria, and apply the approach to discover that inclusion of phosphatase inhibitors in the preparation of the heart mitochondria results in a specific decrease in rates of Complex I-dependent respiration. We believe this new technique will be particularly useful for drug screening and for generating previously unobtainable respiratory data on small mitochondrial samples.

## Introduction

Enhanced appreciation of the role of altered mitochondrial function in metabolic and cardiovascular disease, tumorigenesis, aging and degenerative diseases, and cell signaling has stimulated the development of a variety of new approaches for the assessment of mitochondrial function [1]–[4]. As the field has moved rapidly toward the discovery of mitochondrial-related molecular mechanisms underlying disease, as well as drugs to prevent or reverse disease development [5]–[12], the demand for more flexible and higher throughput methods of assessing mitochondrial function has increased. As well, the importance of screening potential drug candidates for mitochondrial toxicity is being recognized [13]. Measurement of rates of O_2_ consumption are extremely valuable in this regard, as electron transport and oxidative phosphorylation reflect the concerted function of both the mitochondrial and nuclear genomes to express functional components of oxidative phosphorylation. In addition, intact cell respiration reflects the influence of multiple hormonal effects, regulated transporters and pathways, and signaling cascades, and is a telling measure of the overall health of cells, particularly due to the susceptibility of mitochondria to oxidative injury.

In recent years, a number of methodologies have been developed to enable more efficient and higher throughput acquisition of O_2_ consumption data [1]–[2], [4]. Of these, the Seahorse XF24 Analyzer was developed to assay cultured cells in a conventional microplate format [4], representing a significant advance in throughput for assessment of cell monolayers rather than cell suspensions as typically done with conventional Clark electrode-based methods.

There are strengths and weaknesses of measurements of intact cell respiration versus isolated mitochondria. The rate of oxygen consumption by intact cells reflects a complex interplay of biological parameters, including the rates of energy demand and production, as well as the nature, availability, and transport of oxidizable substrates, the effects of signaling cascades that impinge on mitochondrial function, and the overall mass/volume of mitochondria per cell. With intact cells, the endogenous rate of respiration can be measured, as well as state 4_o_ (resting respiration in the presence of oligomycin) and uncoupler-stimulated respiration. However, an observed change in the rates of respiration of intact cells (e.g. as a function of treatment with a drug or altered expression of a gene of interest) can be somewhat difficult to interpret. A change in intact cell respiration may owe to multiple potential alterations that cannot be distinguished without further experimentation, including the rate of ATP utilization, and the transport, storage and mobilization of added and endogenous substrates. As a result, it is often desirable and most informative to also collect respiratory data with isolated mitochondria and thus be able to control the availability of substrates and ADP. Assays with isolated mitochondria allow more direct determination of the potential site of action of a compound or gene product that affects mitochondrial bioenergetics. Further, there are many instances in which valuable information can be obtained from characterizing mitochondria isolated from a limited amount of tissue, for instance, from tissues of transgenic or knockout animal models, or animals in which tissue-specific toxicity of drug candidates need to be characterized.

As a result, we focused our efforts on developing an assay using isolated mitochondria in the XF24 analyzer, and have successfully devised a protocol that allows measurement of mitochondrial O_2_ consumption with as little as 1 µg of mitochondrial protein per well in a multi-well format, facilitating the quantity of information and minimizing the time it takes to gather respiratory data from small tissue samples.

## Methods

### Materials

Fatty acid-free BSA and a protein phosphatase inhibitor cocktail (Phosphatase Inhibitor Cocktail Set II) were purchased from EMD Biosciences. Cell-Tak® was purchased from BD Biosciences. Purified H_2_O purchased from Thermo Scientific was used for respiratory media and reagents. Bradford Assay reagent was purchased from Bio-Rad. All other chemicals were purchased from Sigma-Aldrich.

### Reagent and Solution Preparation

Mitochondrial isolation buffer (MSHE+BSA) is composed of 70 mM sucrose, 210 mM mannitol, 5 mM HEPES, 1 mM EGTA and 0.5% (w/v) fatty acid-free BSA (pH 7.2). Mitochondrial assay solution (MAS, 1X) comprises 70 mM sucrose, 220 mM mannitol, 10 mM KH_2_PO_4_, 5 mM MgCl_2_, 2 mM HEPES, 1 mM EGTA and 0.2% (w/v) fatty acid-free BSA, pH 7.2 at 37°C. A 2–3X stock of MAS was prepared for dilution of substrates, ADP and respiration reagents. Stocks of succinate, malate, glutamate, pyruvate (0.5 M) and ADP (1 M) were made in H_2_O and adjusted to pH 7.2 with potassium hydroxide. Stocks of 10 mM FCCP [carbonyl cyanide 4-(trifluoromethoxy)phenylhydrazone], 2 mM rotenone, 5 mg/ml oligomycin and 40 mM antimycin A were made in 95% ethanol. All reagents were stored at −20°C, except pyruvate, which was prepared fresh on the day of each experiment.

### Isolation of Mouse Liver Mitochondria

Ethics Statement: Animal housing, euthanasia, and tissue harvest procedures were conducted in accordance with and approved by the UCSD Institutional Animal Care and Use Committee (protocol #S09186) and the Buck Institute Animal Care Committee (protocol #10180). Mitochondria from C57bl/6 (male and female) mice aged 4–6 weeks were isolated by two similar differential centrifugation methods, based upon Schnaitman and Greenawalt [14] or Chappell and Hansford [15]. Specifically, the liver was extracted and minced in ∼10 volumes of MSHE+BSA (4°C), and all subsequent steps of the preparation were performed on ice. The material was rinsed several times to remove blood. The tissue was disrupted using a drill-driven Teflon glass homogenizer with 2–3 strokes. Homogenate was centrifuged at 800 g for 10 min at 4°C. Following centrifugation, fat/lipid was carefully aspirated, and the remaining supernatant was decanted through 2 layers of cheesecloth to a separate tube and centrifuged at 8000 g for 10 min at 4°C. After removal of the light mitochondrial layer, the pellet was resuspended in MSHE+BSA, and the centrifugation was repeated. The final pellet was resuspended in a minimal volume of MSHE+BSA. Total protein (mg/ml) was determined using Bradford Assay reagent (Bio-Rad). Typically, ∼7.5 mg of mitochondria (100 µl volume) was obtained from a single mouse liver. In separate studies in which respiratory rates in the Seahorse and the Rank Clark electrode system were compared, mouse liver mitochondria were isolated according to Chappell and Hansford [15] in 250 mM Sucrose, 5 mM Tris and 2 mM EGTA (STE) on ice. Tissue was homogenized 10 times with a Teflon-glass homogenizer, and the homogenate was centrifuged at 1000 g for 3 minutes (4°C). The supernatant was collected and centrifuged at 11,600 g for 10 minutes. The pellet was resuspended in STE after discarding the whitish layer. The above step was repeated two times to get the final mitochondrial pellet. 8–10 mg of mitochondrial protein was obtained from each mouse liver and resuspended in 400–500 µl of STE.

### Isolation of Rat Heart Mitochondria and Phosphatase Inhibitor Treatment

Ethics Statement: Animal housing, euthanasia, and tissue harvest procedures were conducted in accordance with and approved by the UCSD Institutional Animal Care and Use Committee (protocol #S09184). Hearts were harvested from adult male (approx. 300 g) Sprague Dawley rats. Mitochondria from two hearts were isolated by differential centrifugation similar to Sordahl [16]. The tissue was minced and then disrupted with a polytron (IKA Works, Wilmington, NC) at 4°C in MSHE containing 0.5% BSA. The disrupted tissue was centrifuged at 27,000 g for 10 min. The pellet was resuspended and centrifuged at 500 g, and the supernatant filtered through cheesecloth and then centrifuged at 10,000 g. The mitochondrial pellet was resuspended and the centrifugation was repeated. The final pellet was resuspended in a minimal volume of MSHE+BSA. Typically, ∼2.5 mg of mitochondria (∼50 µl volume) was obtained from two rat hearts. Where indicated, the minced tissue was split into two aliquots. To one aliquot, a 1∶400 dilution of phosphatase inhibitor cocktail Set II (EMD Chemicals, Cat. No. 524625) was included in the MSHE+BSA for the remainder of the preparation. At this dilution, the final concentrations of phosphatase inhibitors present in the isolation medium were 0.5 mM imidazole, 0.25 mM sodium fluoride, 0.3 mM sodium molybdate, 0.25 mM sodium orthovanadate, and 1.0 mM sodium tartrate.

### Clark Electrode Assays

Clark electrode assays performed for comparative purposes utilized a Hansatech Oxytherm apparatus (PP Systems, Amesbury, MA) for rat heart mitochondria or a Rank system (Rank Brothers, Bottisham, Cambridge, England) for mouse liver mitochondria. For rat heart mitochondria, assays were performed in parallel with the same mitochondrial preparation, MAS, substrates and compounds as for the XF24 assays. Typically 62.5–125 µg of mitochondria were used in a volume of 500 µl MAS plus the appropriate substrate. Respiration was initiated by adding mitochondria, and followed by sequential addition of ADP, oligomycin and FCCP. Concentrations of substrate, ADP, oligomycin, and FCCP were identical to those used in the XF24 experiments. For mouse liver mitochondria, assays were performed in parallel the same mitochondrial preparation, MAS, substrates and compounds as for the XF24 assays with the following modifications: substrate was 5 mM succinate, 2 µM rotenone and 300 µM ADP was used. Typically, 0.3 mg/ml of mitochondria were used in a volume of 2.0–3.5 ml MAS plus the appropriate substrate. Respiration was initiated by adding mitochondria, followed by sequential addition of ADP, oligomycin and FCCP. Concentrations of oligomycin and FCCP were identical to those used in the XF24 experiments. Oxygen consumption rates were converted from nmol O/min/ml to pmol O_2_/min/µg mitochondrial protein.

### The XF assay using isolated mitochondria

All XF assays were performed using an XF24–3 Extracellular Flux Analyzer (Seahorse Bioscience). The assay is based upon fluorimetric detection of O_2_ and H^+^ levels via solid state probes on a sensor cartridge that lowers to within 200 microns of the well bottom during a measurement cycle, creating a transient micro chamber (∼7 µl) [4]. After a measurement cycle, the sensor cartridge rises, and the medium is re-oxygenated through mechanical mixing, thus allowing repeated measurements of O_2_ and pH over time. The sensor cartridge is equipped with four reagent delivery chambers per well for injecting compounds into the wells during an assay. Either rates of O_2_ consumption (OCR, oxygen consumption rate in pmoles O_2_/min) and changes in pH, or absolute levels of O_2_ and pH can be visualized in the data output.

**Figure 1:**
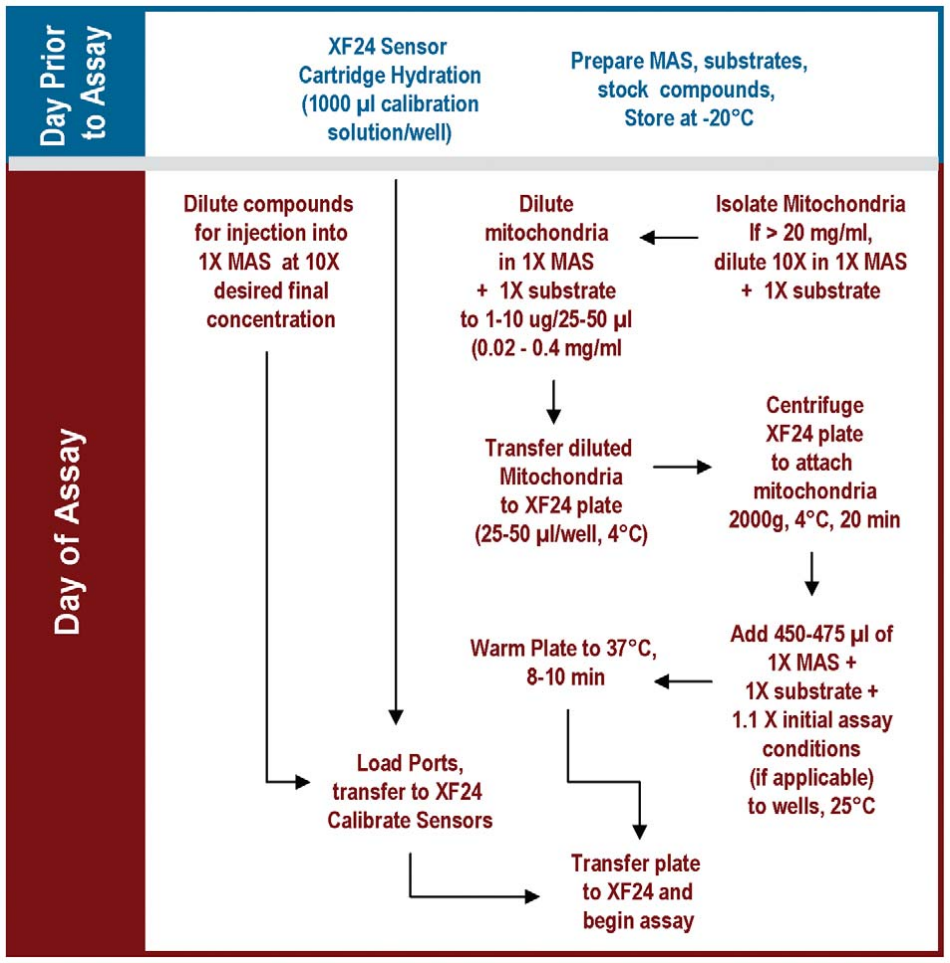
Schematic flowchart for the isolated mitochondria assay using the Seahorse XF24 Analyzer. Mitochondria are diluted into 1X MAS containing the substrate of choice. Initial conditions refer to any additives or compounds present at 1X at the start of the assay in addition to the substrate (e.g. drug candidate, etc.).

A schematic diagram of the assay is presented in Fig. 1. Compounds to be injected were prepared in 1X MAS at 10X the final concentration required for the assay. Compounds were loaded into the injection ports at the following volumes: Port A, 50 µl; Port B, 55 µl; Port C, 60 µl and Port D, 65 µl, which yields an ∼10X dilution for each injection. Just before attachment of the mitochondria to the XF plate, the loaded cartridge was placed into the XF24 instrument and calibrated.

To minimize variability between wells, mitochondria were first diluted 10X in cold 1X MAS + substrate, then subsequently diluted to the needed concentration required for plating. Next, 25 or 50 µl of this mitochondrial suspension was delivered to each well (except for background correction wells) while the plate was on ice. Note that substrate was included in the initial dilution and was present during centrifugation, as this improved the respiratory control ratios obtained in the assay. Centrifugation in larger volumes of mitochondrial suspension resulted in lower maximal respiratory rates, likely due to loss of mitochondria to the sides of the wells. The plate was transferred to a centrifuge equipped with a swinging bucket microplate adaptor, and was spun at 2000 g for 20 minutes at 4°C. After centrifugation, 450 or 475 µl of 1X MAS + substrate (at room temperature) was added to each well. The mitochondria were viewed briefly under a microscope at 20X magnification to ensure consistent adherence to the well, then placed at 37°C for 8–10 minutes to allow the plate to warm. The plate was then transferred to the XF24 instrument and the experiment initiated. For comparison of adhesion with polyethyleneimine (PEI) and Cell-Tak®, plates were coated with PEI as described in Gerenscer *et al* [17] and plates were coated with Cell-Tak® per manufacture's instructions.

### Experimental Design

Two types of experiments are presented with isolated mitochondria. In the first, respiration by the mitochondria (5 µg/well) was sequentially measured in a coupled state with substrate present (basal respiration), followed by State 3 (phosphorylating respiration, in the presence of ADP and substrate), State 4 (non-phosphorylating or resting respiration) following conversion of ADP to ATP, State 4_o_ (induced with the addition of oligomycin), and then maximal uncoupler-stimulated respiration (State 3_u_). This allows respiratory control ratios (RCR; State 3/State 4_o_, or State 3_u_/State 4_o_) to be assessed [18]–[20]. Unless otherwise noted, the substrate was 10 mM succinate plus 2 µM rotenone. Injections were as follows: port A, 50 µl of 40 mM ADP (4 mM final); port B, 55 µl of 25 µg/ml oligomycin (2.5 µg/ml final); port C, 60 µl of 40 µM FCCP (4 µM final); and port D, 65 µl of 40 µM antimycin A (4 µM final). The second type of experiment examined sequential electron flow through different complexes of the electron transport chain. With the initial presence of 5 µg mitochondria per well, 10 mM pyruvate, 2 mM malate and 4 µM FCCP, injections were made as follows: port A, 50 µl of 20 µM rotenone (2 µM final); port B, 55 µl of 100 mM succinate (10 mM final); port C, 60 µl of 40 µM antimycin A (4 µM final); port D, 65 µl of 100 mM ascorbate plus 1 mM N,N,N′,N′-Tetramethyl-p-phenylenediamine (TMPD, 10 mM and 100 µM final, respectively). Typical mix and measurement cycle times for the assays are illustrated in Table 1 and are common to all experiments presented unless otherwise noted.

**Table 1:** Mix and Measure Cycle Times.

Command	Time (min)	Port
Calibrate		
Mix	1.0	
Wait	3.0	
Mix	1.0	
Wait	3.0	
Mix	0.5	
Measure	3.0	
Mix	1.0	
Measure	3.0	
Mix	0.5	
Inject		A
Mix	0.5	
Measure*	3.0–6.0	
Mix	1.0	
Inject		B
Mix	0.5	
Measure	3.0	
Mix	1.0	
Inject		C
Mix	0.5	
Measure	3.0	
Mix	1.0	
Inject		D
Mix	0.5	
Measure	3.0	

*Measure times for State 3 respiration may be extended beyond 3 min to observe the transition from State 3 to State 4 due to exhaustion of ADP in the microchamber.

### XF data treatment

**Figure 2:**
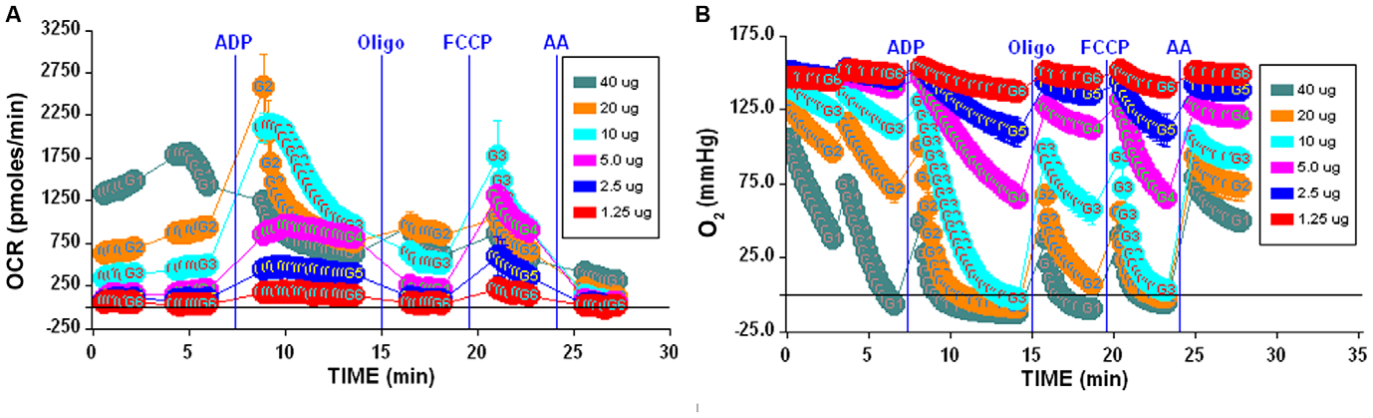
Optimization of isolated mitochondria XF assays. 2A–B, Determination of optimal µg amount of mitochondria/well. 1.25–40 µg/well of mouse liver mitochondria were attached to a V7 polystyrene XF24 plate and the coupling experiment was performed as described in Methods in the presence of succinate/rotenone. Blue vertical lines denote injections of indicated compounds. 2A shows OCR for 1.25–40 µg samples. 2B shows the absolute O_2_ tension (in mm Hg) in the microchamber for 1.25–40 µg samples. Note that samples at 10 µg and above show unstable State 3 rates for OCR and depletion of O_2_ in the microchamber in panels A and B, respectively. Lettering within data points indicates the group identification number.

**Figure 3:**
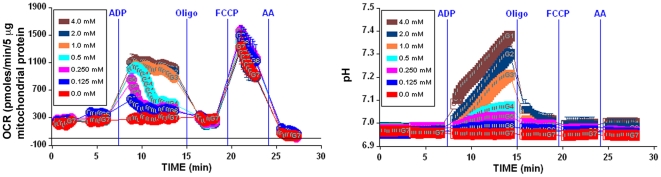
Characterization of mitochondrial activity. 3A, Titration of ADP using 5 µg mouse liver mitochondria/well. ADP (0–4 mM) was injected via port A to initiate State 3 respiration and the measurement time was extended to 6 minutes. Note that 2–4 mM ADP is sufficient to maintain a relatively stable State 3 respiration rate for the duration of the measurement period, while lower concentrations show exhaustion of ADP and transition to State 4 respiration. 3B, Alkalinization of the media during phosphorylating respiration (note that unlike the OCR tracings, this data reports absolute pH rather than a rate of change in pH).

**Figure 4:**
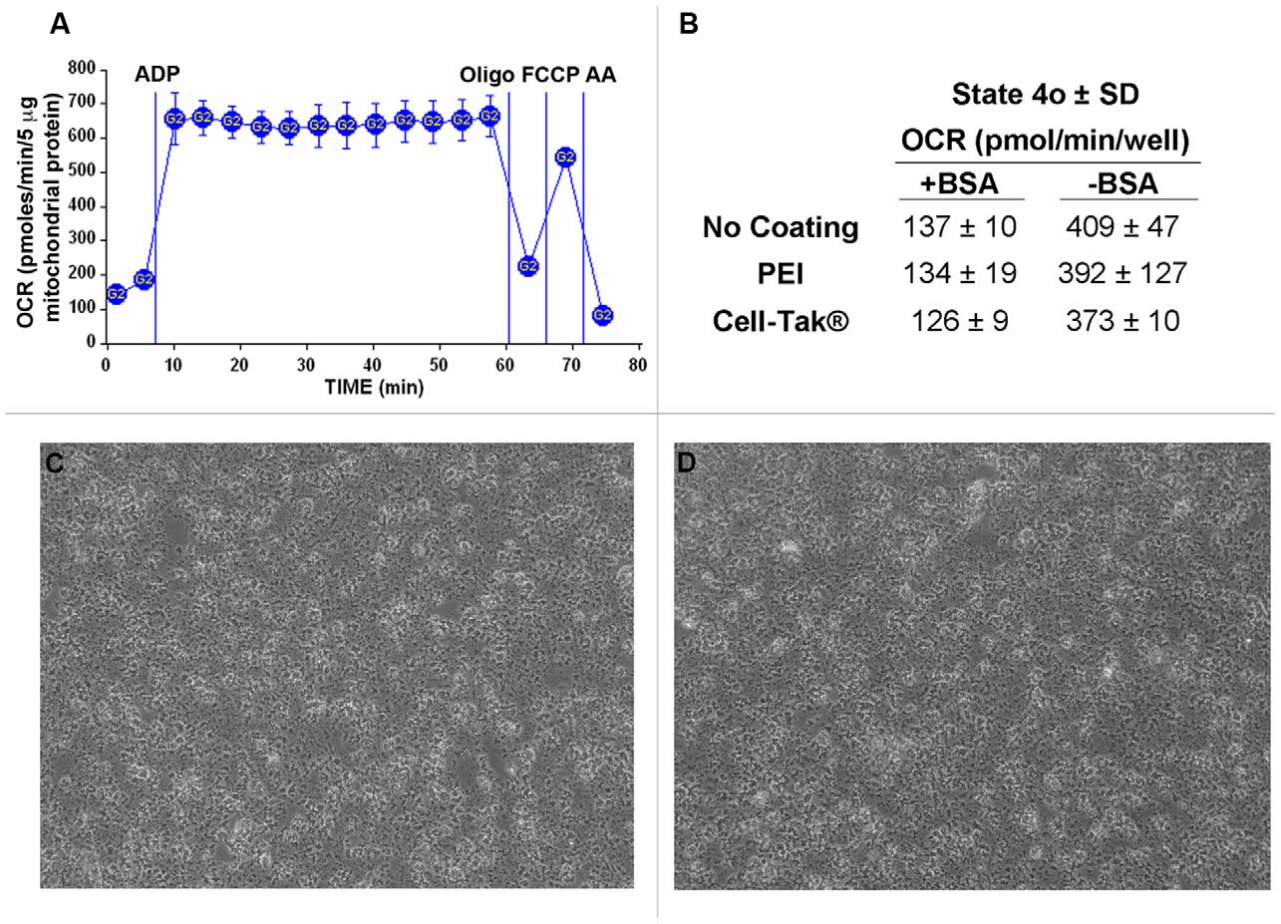
Isolated mitochondria remain attached to the plate for the duration of the experiment. A, The coupling experiment was performed using 5 µg mouse liver mitochondria per well as described in Methods, however, State 3 respiration was allowed to proceed for multiple measurement periods (average OCR per measurement period shown). Note that State 3 respiration does not diminish over multiple mixing and measuring periods, indicating that the mitochondria remain attached to the well for the duration of the assay. B, State 4_o_ rates using different plate coatings in the presence and absence of 0.2% BSA. No significant differences in State 4_o_ rates were observed among different plate coatings [none, polyethyleneimine (PEI), and Cell-Tak®]. Note that the absence of BSA resulted in elevated rates of State 4_o_ respiration, indicative of respiratory uncoupling. C–D, Isolated mitochondria adhered to the XF24 plate as imaged by phase contrast microscopy at 20X magnification before (4C) and after the XF assay (4D).

XF oxygen consumption rate (OCR) raw data was transformed using the “Level(Direct)Akos” algorithm that is a component of the Seahorse XF24 1.5.0.69 software package. This algorithm is fully described in Gerencser *et al* [17]. The software may be configured to show kinetic data in a “point-to-point” or “middle point” mode. The point-to-point mode (e.g. Figs. 2, 3) displays OCR as a series of rates across the measurement period and can show changes of the rate across the measurement period (e.g. Fig 3A). The middle point mode shows a single OCR value for the measurement period, and is the average of the point-to-point rates (e.g. Fig. 4A) Note that if the point-to-point rates are stable (relatively constant) across the measurement period, then point-to-point and middle point modes will provide equivalent rate data. All experiments described and shown were repeated at least 3 times with similar results based on respiration rates and RCR values obtained. Figures presented are representative graphs of the illustrated assay.

## Results

### Validation of the XF method

The XF24–3 Analyzer is a 24 well microplate-based instrument that was designed to measure in real time the kinetics of O_2_ consumption and H^+^ production of a monolayer of cells attached to the wells of an XF24 microplate. Our aim in these studies was to find conditions that might allow use of the XF24 with isolated mitochondria. Following the successful introduction of centrifugation as a means to attach suspended synaptosomes to XF24 microplates [21], we used the same approach to attach mitochondria to the plate by centrifugation and measured OCR. We ultimately identified conditions that allowed for sequential measurement of basal respiration (in the presence of substrate but no ADP), State 3 (+ADP), State 4_o_ (+oligomycin), and State 3_u_ (+FCCP) by the protocol described in Fig. 1, and these differ significantly from typical conditions for conventional O_2_ electrode measurements [22]. Table 1 outlines the general mix and measure cycle times used for the assays. The results using 5 µg mitochondrial protein per well in Fig. 2A (5 µg group) show the optimal result: a steady low rate of respiration with substrate before addition of ADP; a substantial increase in rate to a high sustained state 3 rate after addition of ADP; a substantial decrease in rate to a low state 4_o_ rate after addition of oligomycin; a high stimulation by FCCP to state 3_u_, and strong inhibition to a near zero rate after addition of the complex III inhibitor, antimycin A (AA).

In Fig. 2, determination of an optimal quantity of mitochondria per well is demonstrated, with the objectives to ensure robust signal, minimal noise, as well as keeping OCR values within the linear range of response of the mitochondria and within the dynamic range of the instrument and algorithms employed to calculate respiration rates. Increasing amounts of mouse liver mitochondria from 1.25–40 µg per well were attached to a plate as described in the Experimental section and basal respiration, States 3, 4_o_, 3_u_, and non-mitochondrial O_2_ consumption were sequentially measured. Basal rates of respiration were linear from 1.25 to 5 µg. The sequentially measured rates as ADP, oligomycin, FCCP, and antimycin A were injected into the wells were generally linear with respect to µg mitochondria used per well. However, for 10 µg or greater per well, the response of the mitochondria for various respiration states became non-linear (note 10, 20 and 40 µg amounts in Fig. 2A). To explain this behavior, the O_2_ concentration (oxygen tension as measured in mm Hg) values were reviewed (Fig. 2B). These data illustrate the result of overloading the wells, and show that with mouse liver mitochondrial samples of 10 µg or greater per well, a) O_2_ can be completely depleted from the microchamber (0.0 O_2_ tension), and b) the system does not have an adequate time to recover to normoxia (return to ambient O_2_ tension, ∼158 mm Hg) before the next measurement cycle. These two factors prevent accurate measurement of OCR at higher concentrations of mitochondria, with State 3 being underestimated and apparent poor response of the mitochondria to oligomycin and FCCP. Thus, when adapting this method to mitochondria isolated from other species/tissues, it is critical to ensure that an optimal amount of mitochondria are used per well. It is suggested that basal respiratory rates be kept between 100–200 pmol/min/well to afford the best signal-to-noise ratio and dynamic range for the assay, and we find that, depending on tissue and species used, 1–10 µg of isolated mitochondria is optimal for the assay.

Our initial use of typical concentrations of ADP (∼200 µM) was unsuccessful at eliciting State 3 rates. Instead, much higher concentrations of ADP were required to obtain stable State 3 rates. This is due to the very small effective volume of the microchamber (∼7 ul) during the measurement cycle (see Discussion). To investigate the effects of ADP concentration, the measurement time for State 3 respiration was extended to 6 minutes and ADP was titrated from 0.0 to 4.0 mM. The point-to-point rate data in Fig. 3A clearly reflects the exhaustion of ADP at lower concentrations (0.25–1.0 mM). Thus, the transition from State 3 to State 4 respiration upon conversion of the pool of ADP to ATP is evident (Fig. 3A), and therefore a respiratory control ratio as defined by State 3/State 4 can be calculated. If a stable State 3 rate is desired, 2–4 mM ADP is required to sustain State 3 respiration over a measurement period of 6 minutes. Note that the decrease in rate at lower ADP concentrations is not the result of O_2_ exhaustion in the microchamber (Fig. 3A versus Fig. 2B, 5 µg). In addition, experiments in which the concentration of phosphate was varied, 10 mM proved optimal and ensures a saturating concentration of phosphate for ATP synthesis (data not shown). Thus, the method provides the ability to record State 3 respiration followed by exhaustion of ADP to State 4 given an appropriate length of measurement cycle.

Another interesting aspect of the assay is the ability to monitor changes in pH. In conventional use of the XF24, changes in extracellular pH act as an indirect monitor of lactate production via glycolysis [4], [23]–[24]. Viewing the correlating pH data from the ADP titration (Fig. 3D), it is observed that an increase in pH occurs during the phosphorylation reaction (after ADP injection) that is quickly abrogated upon the addition of oligomycin. This reflects that the ATP synthesis reaction consumes protons as the ADP is phosphorylated to ATP: MgADP**^−^** + P_i_^−1.5^ +0.5H**^+^→** MgATP^−2^ + H_2_O (assuming P_i_ is an approximate 50∶50 mixture of P_i_^−1^ and P_i_^−2^ at neutral pH).

A critical element of the method was to ensure that the mitochondria adhere tightly to the wells and do not detach from the plate during the mixing action of the instrument over the course of the assay. While this has been previously demonstrated using polyethyleneimine (PEI) as a surface coating [17], we wished to test if a plate coating was necessary, or if other coatings may be more optimal for adhesion. Four distinct methods were employed to investigate adherence of the mitochondria to the well plate. First, it was assessed whether repeated measurement cycles, each associated with a mixing/reoxygenation step, resulted in a loss of mitochondria and therefore a loss in respiratory rate. Figure 4A (no plate coating used) illustrates via repeated measurements of State 3 respiration (12 repeated measurements over ∼50 minutes) that the ADP-stimulated rates remain consistent throughout the assay, strongly suggesting that mitochondria remained attached to the well for the duration of the assay. Note that each point shows the average OCR for each 3 minute measurement cycle (as opposed to point-to-point rates, see “data treatment” in the Methods section). Second, experiments were performed with no coating, or plates coated with PEI or Cell-Tak®. Coating with Cell-Tak® or PEI did not alter State 4_o_ rates compared to rates with no coating used (Fig. 4B). Third, phase contrast images of the same well immediately before and after the experiment indicated that there were no noticeable differences in the appearance or density of the mitochondria, indicating that the mitochondria remained tightly attached to the well plate (Fig. 4C and D, no plate coating used).

Experiments that measured any potential release of total mitochondrial protein during the assay were performed by comparing the total protein concentration in the supernatant of replicate wells before and after the experiment. These parallel assays were performed in the absence of BSA to allow accurate quantification of small concentrations of protein. Greater than 95% of the total mitochondrial protein was adhered to the plate before the experiment, and <10% of total adhered protein was detectable in the supernatant after the assay (data not shown).

It was also determined that mouse liver mitochondria required the presence of BSA in the assay medium to prevent mitochondrial uncoupling (Fig. 4B). State 4_o_ rates were significantly higher in the absence of BSA, and further experiments testing a range of 0.0 to 0.8% BSA indicated that 0.2% BSA is required to obtain minimal basal and State 4_o_ rates (data not shown).

**Figure 5:**
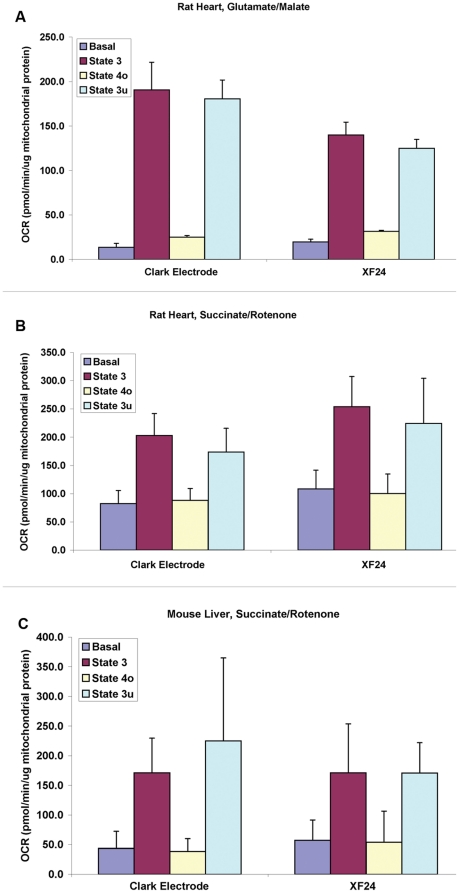
Comparison of Clark electrode and XF technology shows comparable respiration data between the methods. Mitochondria isolated from rat heart and mouse liver were used in parallel coupling experiments using either a Hansatech or Rank Clark type electrode or the XF24. Assays were performed as described in Methods for each platform, respectively. Comparison of Basal, State 3, State 4o and State 3u rates between the Hansatech and XF with rat heart using or glutamate/malate as substrate (5A) or succinate/rotenone (5B), respectively. Comparison of Basal, State 3, State 4_o_ and State 3_u_ rates between the Rank and XF with mouse liver mitochondria using succinate/rotenone (5C). Data are expressed as mean ± SD from 3 separate experiments in Fig 5A and B, and mean ± SD from 4 experiments in 5C. The high SD in 5C owes to higher rates obtained with one of the four mouse liver preps, rather than variation between methodologies on a given day. Data were analyzed using a two-factor ANOVA with repeated measures on one factor. An interaction was detected only in the data of Fig. 5A, and post-hoc paired comparisons detected lower rates in the XF24 of State 3 and 3_u_, and a higher rate of State 4_o_ respiration with rat heart mitochondria oxidizing glutamate and malate (p<0.05).

We next compared the rates of mitochondrial respiration obtained from experiments in the XF24 to those obtained in more conventional measurements of oxygen consumption using a Clark-type electrode. Respiratory rates (basal, State 3, State 4_o_, and State 3_u_) of isolated rat heart mitochondria oxidizing glutamate/malate (Fig. 5A) or succinate with rotenone (Fig. 5B) measured in the XF24 were in a similar range to those obtained using a Hansatech Oxytherm apparatus under similar conditions (37°C in MAS buffer). In addition, respiration rates of isolated mouse liver mitochondria were comparable using the XF24 and a Rank system (Fig. 5C).

### Applying the XF method

**Figure 6:**
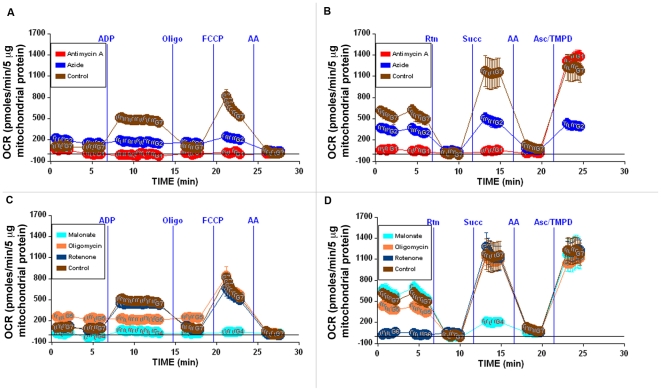
Using the Coupling and Electron Flow assays in tandem to elucidate mechanistic activity of test agents. Coupling (A, C) and electron flow experiments (B, D) were performed as described in Methods. Initial conditions are as follows (with final concentrations listed): A–B, Controls (no additives) or 20 mM sodium azide or 4 µM antimycin-A; C–D, Controls (no additives) or 10 mM malonate or 2.5 µg/ml oligomycin or 2 µM rotenone. See text for further explanation of results.

To demonstrate the potential utility of this method for determining the effects of agents on mitochondrial respiration, a series of experiments were conducted examining 1) the level of respiratory coupling (the ‘coupling experiment') and 2) the sequential determination of complex I, II, III and IV-dependent respiration (the ‘electron flow experiment'). Fig. 6 demonstrates the effects of known respiratory inhibitors on the pattern of respiration. For the coupling experiment (Fig. 6A, C), mouse liver mitochondria were incubated with substrate (10 mM succinate +2 µM rotenone) during the centrifugation step. ADP, oligomycin, FCCP, and antimycin A were then sequentially injected, and measurements of OCR were taken after each injection. The electron flow experiment (Fig. 6B, D) is designed to follow and interrogate each complex of the electron transport chain. This experiment begins with the mitochondria utilizing Complex I respiration in an uncoupled state (10 mM pyruvate, 2 mM malate plus 4 µM FCCP, final concentrations). The following compounds (final concentrations) are then added sequentially: rotenone (2 µM), succinate (10 mM), antimycin A (4 µM) and ascorbate/TMPD (1 mM and 100 µM, respectively). Because oxidation of pyruvate/malate is mediated via Complex I, injection of rotenone inhibits this and respiration stops. Injection of succinate allows the mitochondria to respire via Complex II, and OCR values increase. Electron flow is then inhibited at Complex III by antimycin A, and respiration stops as expected. Finally, addition of ascorbate and TMPD (which act as electron donors to cytochrome C/complex IV) elicits an increase in the OCR.

By using these two assays in tandem in a single microplate, it now becomes possible to pinpoint sites of action of unknown compounds (e.g. potential drug candidates) on mitochondrial function, and this data is presented in Fig. 6A–D. To illustrate the method, we have chosen five well-described compounds that affect mitochondrial function to represent the “unknown compounds”. Each of these compounds was added to the wells as “initial conditions” when the additional 450 µl of MAS + substrate was added after the centrifugation step (Fig. 1).

Figure 6C–D show the effects of rotenone (a Complex I inhibitor) on coupling and electron flow. As expected, there is no effect on the OCR values in the coupling experiment, as rotenone was already present and respiration was being driven by Complex II-IV activity. However, it was observed in the electron flow experiment that pyruvate/malate-dependent respiration was inhibited at the beginning of the assay in contrast to the control, in which robust respiration was present. The fact that the control and the group to which rotenone was added show identical subsequent responses upon injections B–D suggests, as anticipated, that the remainder of the electron transport chain is functioning properly.

The effects of including malonate or antimycin A (Fig. 6, panels C–D or A–B, respectively) in the initial conditions of coupling and electron flow assays are shown. Malonate is a competitive inhibitor of succinate dehydrogenase (complex II), and antimycin A inhibits complex III. For malonate, as anticipated, all the respiratory rates are inhibited except for the respiration driven by pyruvate/malate at the start of the electron flow experiment, and the ascorbate/TMPD-driven rate mediated by complex IV. Like malonate, the effect of antimycin A in the coupling experiment was complete respiratory inhibition and no response to ADP, oligomycin, or FCCP. However, unlike malonate, antimycin A prevents both complex I- and complex II-mediated respiration due to inhibition of complex III, resulting in complete inhibition of respiration throughout the electron flow portion of the assay until addition of ascorbate/TMPD, indicating that complex IV remains active.

Figure 6, panels A–B or C–D show the effects of the azide anion or oligomycin on respiration, respectively. Azide, like carbon monoxide and cyanide, is an inhibitor of complex IV. This is demonstrated in both the coupling and electron flow experiments, with reduced respiration throughout the assay. Note that while complete inhibition is not observed due to sub-saturating concentrations of azide, overall respiration is decreased. Most instructive is the fact that addition of ascorbate/TMPD could not increase electron flow (and in turn O_2_ consumption) at Complex IV. Finally, oligomycin, an inhibitor of the ATP synthase (complex V) prevents only ATP synthesis (the ADP-stimulated rate, State 3) in the coupling experiment, but does not affect electron flow through the complexes under uncoupled conditions.

**Figure 7:**
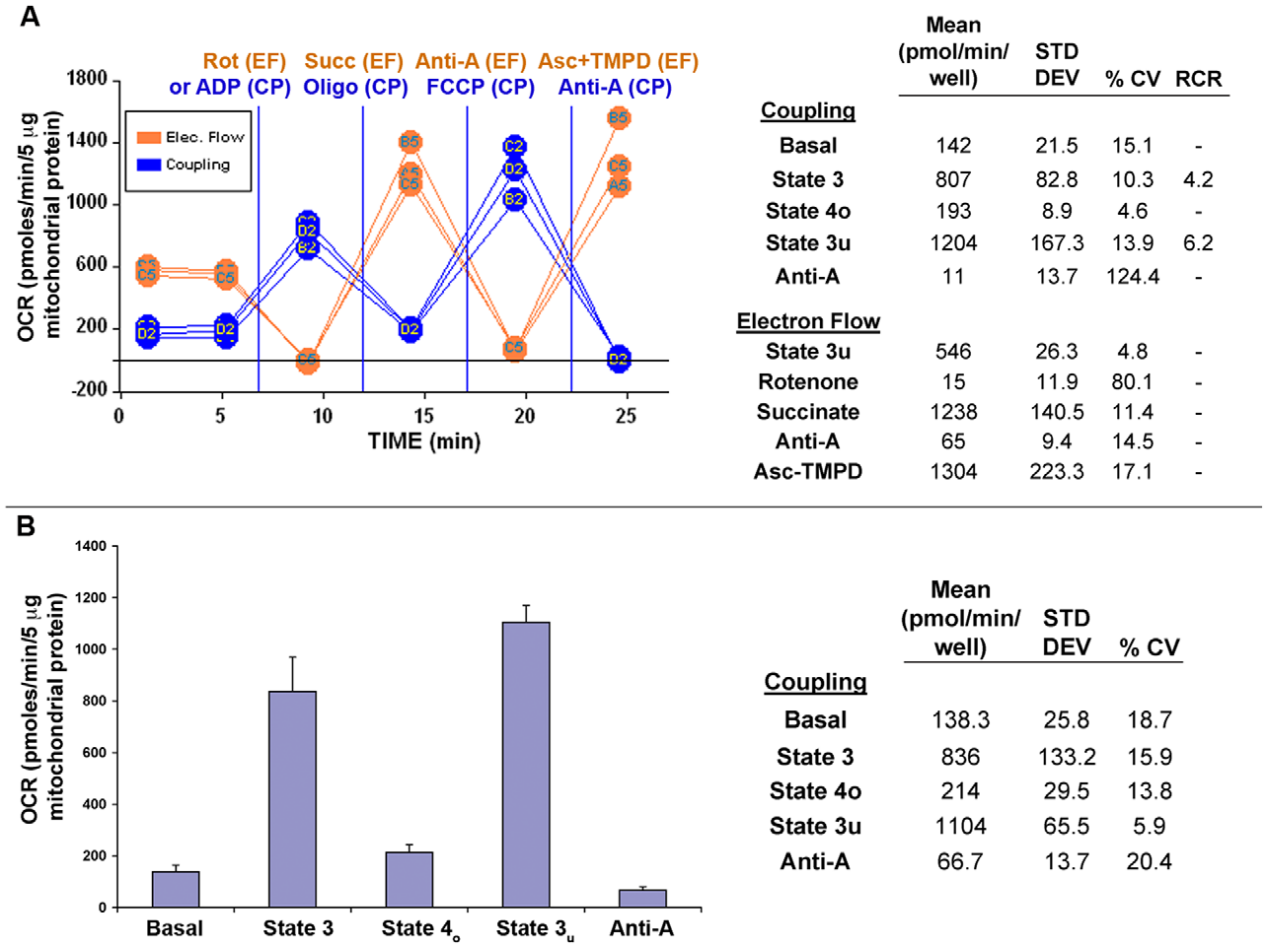
Assay Reproducibility. In 7A, intra-assay reproducibility is demonstrated. Assays used 3–5 replicate wells and well-to-well variation within electron flow and coupling assays shows coefficients of variation (CV) <17% in all measurements (except where OCR has been reduced to minimal levels with rotenone or antimycin-A). In 7B, inter-assay reproducibility is demonstrated. Four separate uncoupling experiments from four different preparations of mouse liver mitochondria were averaged to illustrate reproducibility of the assay over multiple days/preparations and shows CVs <20%. The corresponding table indicates the means, standard deviations and% CV, average RCR values are given in the text.

The coupling and electron flow experiments were also used to demonstrate intra- and inter-assay variability. Typically, 3–5 replicate wells per group were used on a plate. Figure 7A shows average rate data from 3 individual wells of an electron flow and a coupling assay, with mean, standard deviation and% coefficient of variation (CV) presented in the corresponding table. Intra-assay rates are very consistent, with CVs of 10.3% and 4.6% for State 3 and State 4_o_ rates, respectively. RCR values were 4.2±0.6 and 6.2±0.6 for States 3/4_o_ and 3_u_/4_o_, respectively. Basal and State 3_u_ values showed ≤15% CVs. The electron flow assay also exhibited consistent results, with CVs <14%. The high CVs reported for antimycin A (coupling) and rotenone (electron flow) treatment arise from the very low OCR levels. To illustrate inter-assay reproducibility, the average of four different mouse liver mitochondrial preparations (different animals/different days) are presented in Fig. 7B. Rates of basal, State 3, and State 3_u_ were reproducible with low standard deviations (CVs <20%). Expressed in more conventional units of respiration, the State 3 rate of respiration on succinate (836±133 pmol O_2_/min/5 µg mitochondrial protein, Fig. 7B) is 334±54 nmol O/min/mg of mitochondrial protein. The RCR value as calculated by State 3/State 4_o_ over the four independent experiments with succinate as substrate was 3.9±0.6 and RCR as calculated by State 3_u_/State 4_o_ was 5.2±0.6.

**Figure 8:**
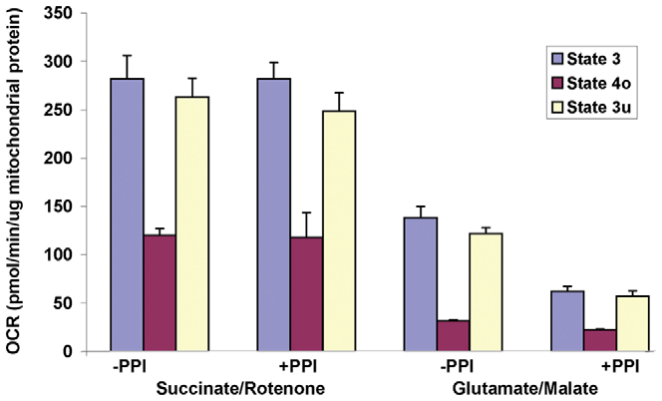
Effects of Phosphatase Inhibitor (PPI) treatment on rat heart mitochondrial respiration. Mitochondria were treated with a cocktail of phosphatase inhibitors during the isolation procedure as described in the Methods. Respiratory States 3, 4_o_ and 3_u_ were measured in the presence (+PPI) or absence (-PPI) of phosphatase inhibitor treatment in the presence of either succinate/rotenone (3 µg/well) or glutamate/malate (6 µg/well) as oxidizable substrates, and rates are expressed per µg mitochondrial protein.

We have additionally applied this methodology to test the effects of a commercially available cocktail of protein phosphatase inhibitors (PPI) on rat heart mitochondrial respiration. The effect of the PPI was tested by adding a 1∶400 dilution of the premixed cocktail to the mitochondrial isolation medium immediately prior to disruption of the tissue with the polytron, and the inhibitors were included throughout the subsequent steps of the isolation, and were included in the MAS during the experiment. At the concentrations used, this cocktail would be expected to inhibit primarily protein tyrosine phosphatases. As indicated in Fig.8, rates of State 3, 4_o_, and 3_u_ respiration with succinate (plus rotenone) were unaffected by PPI treatment. In contrast, rates of glutamate/malate-driven State 3 and 3_u_ were significantly lower as a result of the inhibitor treatment. This approximate 50% reduction suggests that PPI treatment affects either the activity of complex I, or the metabolism or transport of glutamate or malate.

## Discussion

We describe the development of a robust and high throughput assay to measure mitochondrial respiration using the XF24 Analyzer. The major advantage of the method described is the ability to run multiple samples (20) simultaneously using very small quantities of material (1–10 µg per well). As a result, it is now possible to perform types of investigations that require higher throughput (for instance, drug screening and/or characterization) and/or studies in which only a small amount of biological material is available (e.g. a single mouse heart or a human muscle biopsy).

The optimal conditions for performing the assay were not initially obvious, and they differ significantly from conventional procedures using a chamber with a Clark-type oxygen electrode. First, the mitochondria must be captured at the bottom of the XF24 plate as opposed to being in suspension for conventional polarography. Second, significantly higher levels of ADP, substrates, and phosphate must be present to sustain respiratory rates. This difference owes to the small volume of the chamber that is created during the measurement cycle in the XF24 (approximately 7 µl). With 5 µg of mitochondria/well, the protein concentration is 0.7 mg/ml, which is in the range of conditions employed in an electrode chamber (typically 0.25–1 mg/ml depending upon the mitochondrial tissue type). However, the total number of moles of ADP, substrate or phosphate available over the measurement cycle in this small volume is much less than a typical chamber of larger volume. For instance, the total quantity of ADP available during the measurement using 0.5 mM ADP in the assay medium is only 3.5 nanomoles. If the State 3 rate of respiration of the mitochondria is 836 pmoles/min (Fig. 6B), and provided that the P:O ratio is approx 1.3 with succinate as substrate [25], then the mitochondria have only enough ADP to sustain State 3 respiration for approximately 2 minutes, which is evident in the tracing in Fig. 3A. Thus, this small ‘closed’ chamber during measurement accounts for the requirement of high concentrations of substrates, phosphate and ADP.

It was necessary to include BSA in the experimental medium with mouse liver mitochondria, as there was significant uncoupling in its absence (Fig. 4B). This may relate to the length of time that the mitochondria spend at 37°C over the course of the experiment, which may be longer than in a conventional electrode chamber. Such a requirement for BSA has generally been attributed to binding of free fatty acids being liberated as a function of ongoing phospholipase activity. It should be mentioned that the presence of BSA in the experimental media shifts the dose response curve to FCCP (a hydrophobic compound) far to the right, thus requiring higher concentrations of FCCP to reach a maximal state 3_u_ rate than in the absence of BSA. It would be expected that the dose response curve of other hydrophobic compounds in the assay would be similarly affected.

We also did not anticipate that mitochondria would be effectively captured and remain adherent throughout repeated mixing and measurement cycles (Fig. 4). Like other tissue culture plastic, the XF24 plates are plasma treated to create a hydrophilic surface for cell adhesion. Tissue culture cell attachment is initially tenuous, after which cells secrete extracellular matrix proteins for which they express protein receptors that mediate spreading and firm adhesion. The surface charge of mitochondria is negative, largely as a result of the polar head groups of phospholipids [26]. This charge, similar to that of other biological membranes, is apparently sufficient to allow mitochondria to adhere on the XF24 plate, and adhesion appeared to be unaffected by the reagents added from the ports or by depolarization with uncoupler. Surprisingly, we did not observe that coating the XF24 plate with polyethyleneimine (a cation), or Cell-Tak™ (a bioadhesive that is a mixture of polyphenolic proteins from a marine mussel) enhanced the maximal rate of respiration after centrifugation of the mitochondria onto the plate (Fig. 4), thus implying that coating did not increase mitochondrial adsorption. We did find that mouse liver mitochondria that had been washed in a physiological salt-containing buffer did not adhere as well as those isolated exclusively in sucrose/mannitol (data not shown). Thus, the adsorption of salt-washed mitochondria might benefit from the use of an adhesive coating.

The respiratory rates with isolated rat heart and mouse liver obtained with the XF24 using the described methodology were generally comparable to rates obtained in Clark-type oxygen electrode apparatuses (Fig. 5). The respiratory control ratio (state 3/state 4_o_) measured for mouse liver mitochondria at 37°C with succinate as substrate was 3.9±0.6 (Fig. 7B). Most literature values are based upon polarographic experiments performed at either room temperature or at 30°C, which is generally protective to mitochondria compared to 37°C, however, the respiratory rates and RCR values reported here are comparable to other reports of polarographic experiments performed at 37°C [27]–[29]. Therefore, we believe that the method described here can yield similar results to more conventional measures of mitochondrial oxygen consumption. In adapting this approach to mitochondria from other tissues and species, mitochondrial quantity, centrifugation volume, content and concentrations of reagents in the assay medium, concentrations of respiratory modulators (ADP, oligomycin, uncoupler) will need to be optimized. Particular attention should be paid to incubation conditions and the overall length of the experiment if using mitochondria that rapidly decline in functional quality with time after isolation.

In addition to demonstrating how this method could be used to characterize potential toxicity or the mechanism of action of compounds (Fig. 6), we also applied the approach to characterize the effects of a cocktail of protein phosphatases on isolated rat heart mitochondrial respiration (Fig. 8), in which we identified a decreased rate of respiration with glutamate and malate but not succinate as oxidizable substrates. During preparation of this manuscript, a similar observation was reported by Hoppel and colleagues [30]. Since early mass spectrometry-based characterization of the heart mitochondrial proteome [31]–[32], understanding of the complete set of proteins involved in the function and regulation of heart mitochondria has rapidly advanced [33]–[34]. Endogenous phosphorylation of multiple mitochondrial proteins and complexes have been described [35]–[41], and characterization of regulatory post-translational modifications that integrate bioenergetics and cell signaling is an area of active discovery. Here, we demonstrate how the described method could be used to assess functional consequences of manipulation of post-translational modifications in isolated mitochondria. As employed, the mixture of protein phosphatases likely inhibited ongoing protein tyrosine phosphatase activity, and members of the protein tyrosine phosphatase family have been localized to mitochondria [36], [37], [42], [43]. Phosphotyrosine modification has been documented in complex I and in specific steps of the pathway of glutamate/malate oxidation in the TCA cycle [40], [41] that could potentially account for our observed alterations in respiratory rates. Further proteomic analysis would be required to establish how the phosphatase inhibitor treatment employed here altered the phosphorylation status of mitochondrial proteins. As well, the assay described here could be performed with alternative Complex I dependent oxidizable substrates to further identify the locus of the effects of phosphatase inhibitors.

In summary, this report provides details of a powerful and novel approach for monitoring mitochondrial respiration and peri-mitochondrial pH changes in relative high throughput with minute quantities of isolated mitochondria. Such an assay lends itself to drug screening or identification of effects of altered gene expression or *in vivo* treatment on the function of subsequently isolated mitochondria. In fact, preliminary data suggest that the XF96 analyzer can be similarly employed with isolated mitochondria. The basic workflow and assay design is identical, however, as the wells of the XF96 plates are 40% of the surface area of the XF24 well plates, it was observed that optimal quantities of mitochondria to use ranged from ∼0.5–5 µg per well (data not shown). In general, the small amounts of mitochondria required for this new assay make it possible to simultaneously gather information on mitochondria isolated from single organs of a mouse or from cultured cells. Within the same plate, as demonstrated in Fig. 6, effects on different sites in the electron transport chain can be probed, and the mechanism of action of a drug or gene product on oxidation of a variety of substrates (such as pyruvate/malate, fatty acyl carnitine/malate, glycerol-3-phosphate, etc) can be determined simultaneously. The concurrent acquisition of data is advantageous in particular for mitochondria that decline in quality over the course of the experimental day following isolation. Overall, we propose that this new methodology can be a valuable tool for the discovery of mitochondrial-targeted therapeutics and the elucidation of mitochondrial-related cell signaling, as well as pathogenesis in a broad variety of conditions including metabolic,cardiovascular and mitochondrial diseases, neurodegeneration, and cancer biology.
